# Willingness and acceptability of cervical cancer screening among HIV positive Nigerian women

**DOI:** 10.1186/1471-2458-13-46

**Published:** 2013-01-17

**Authors:** Oliver C Ezechi, Chidinma V Gab-Okafor, Per Olof Ostergren, Karen Odberg Pettersson

**Affiliations:** 1 Clinical Sciences Division, Nigerian Institute of Medical Research, Lagos, Nigeria; 2 Division of Social Medicine and Global Health, Faculty of Medicine, Lund University, Lund, Sweden

**Keywords:** Cervical cancer, Screening, HIV, Acceptability

## Abstract

**Background:**

The proven benefit of integrating cervical cancer screening programme into HIV care has led to its adoption as a standard of care. However this is not operational in most HIV clinics in Nigeria. Of the various reasons given for non-implementation, none is backed by scientific evidence. This study was conducted to assess the willingness and acceptability of cervical cancer screening among HIV positive Nigerian women.

**Methods:**

A cross sectional study of HIV positive women attending a large HIV treatment centre in Lagos, Nigeria. Respondents were identified using stratified sampling method. A pretested questionnaire was used to obtain information by trained research assistants. Obtained information were coded and managed using SPSS for windows version 19. Multivariate logistic regression model was used to determine independent predictor for acceptance of cervical cancer screening.

**Results:**

Of the 1517 respondents that returned completed questionnaires, 853 (56.2%) were aware of cervical cancer. Though previous cervical cancer screening was low at 9.4%, 79.8% (1210) accepted to take the test. Cost of the test (35.2%) and religious denial (14.0%) were the most common reasons given for refusal to take the test. After controlling for confounding variables in a multivariate logistic regression model, having a tertiary education (OR = 1.4; 95% CI: 1.03-1.84), no living child (OR: 1.5; 95% CI: 1.1-2.0), recent HIV diagnosis (OR: 1.5; 95% CI: 1.1-2.0) and being aware of cervical cancer (OR: 1.5; 95% CI: 1.2-2.0) retained independent association with acceptance to screen for cervical cancer.

**Conclusions:**

The study shows that HIV positive women in our environment are willing to screen for cervical cancer and that the integration of reproductive health service into existing HIV programmes will strengthen rather than disrupt the services.

## Background

Each year about half a million women develop invasive cancer of the uterine cervix, with more than 80% occurring in low income countries [1,2]. A majority of the cases presents in late stages when available treatments are ineffective [1,3]. The scenario is entirely different in high-income countries where cervical cancer has almost been eliminated as a result of efficient cervical cancer prevention programmes [1,4].

In sub Saharan African countries where cervical cancer is endemic; HIV infection has become one of the leading causes of death in women [5], making the interactions between the diseases a major public health challenge [6]. The risk of developing cervical cancer and increased aggressiveness of existing cervical cancer has been reported in HIV infected women [7,8]. Integrating cervical cancer prevention within HIV care services will not only decrease the morbidity and mortality associated with invasive cervical cancer but will also improve HIV treatment outcomes [5,7,8]. Investigators in Zambia in a report of the evaluation of the success of their integration of cervical cancer prevention services into existing HIV care services concluded that the model be adopted as an implementation platform low-cost cervical cancer prevention [9].

The new Nigerian National HIV treatment guidelines recognizing the potential benefit of cervical cancer prevention in HIV infected women, recommended the introduction of cervical cancer screening within HIV programmes as a standard of care [10]. Unfortunately, this important recommendation is not yet operational in most HIV clinics, either as a result of lack of will to do so or for the fear of possible disruption of the successful HIV services [1,8,11,12]. The latter reason is not backed by scientific evidence, as the only published study from Nigeria on the subject in women of known HIV positive status showed a 96.0% ‘willingness to screen’ in future [13]. However this study was among women who have just completed posttest counseling and not yet enrolled into HIV care, making it difficult to use the information to extrapolate what the situation would be in HIV care and treatment setting. In addition, varying refusal rates have been reported from other high HIV prevalent countries ranging from 12-87% [14-17]. Determining the cervical cancer screening refusal rates among HIV positive Nigerians already in HIV care is therefore necessary as it will not only be useful for the refinement of programmes but as an advocacy tool.

This study was therefore conducted to determine the acceptability of cervical cancer screening among HIV infected women using a sample population that is sufficiently powered to make generalization possible. In addition, the predictors of acceptance of cervical cancer screening among these women were also determined. Information obtained will be used to fine-tune the integration of cervical cancer screening process, which is ongoing in our HIV treatment centre and hopefully in similar clinics in Nigeria and elsewhere.

## Methods

### Study design and setting

The study was a cross-sectional survey conducted at the HIV treatment centre, Nigerian Institute of Medical Research (NIMR), Lagos. NIMR is the apex medical research institution in Nigeria charged with the responsibility to conduct research into disease of public health importance in the Country. However following the initiation of the Federal Government of Nigeria antiretroviral drug access programme in 2002, it was selected as one of the 25 centres. It was selected principally to meet the research component of the programme. The centre currently provides comprehensive HIV care, treatment and support for over 18,000 patients (64.6% are women). Sixty five percent of the patients come from Lagos and the rest from the other 5 states of southwestern Nigeria, North-central, South-south and South-eastern Nigeria. A little over 0.025% comes from the neigbouring western African countries. The services at the centre are provided free to the patients.

### Study population

Adult females aged 18 years and older seen at the centre for their monthly antiretroviral drug refill, 3-6monthly physician appointment or for registration into HIV care from 1st to 30th of April 2011.

### Study sample size determination

The sample size for the study was determined using Raosoft sample size calculator ( http://www.raosoft.com/samplesize.html) [18]. Given that there were approximately 9,000 women who are current on the programme, on the basis of the most conservative response distribution of 50%, allowing 2.5% margin of error at 95% confidence interval, the required sample size was calculated to be 1313. The sample size was increased by 15% in anticipation of non-response as in a previous study in the centre. A final minimum sample size of 1510 was obtained.

### Study sample selection

Participants were approached for the interview after selection from the lists of attendance using proportionate stratified sampling method. This is to enable generalization of the findings of the study to the women at the clinic. Each of the patient’s categories in the clinic of drug refill, 3-6monthly physician consult and new patients were considered to be a homogenous population. These three categories were often in the ratio of 50: 25:1 at each clinic day. Daily list of clinic attendee was categorized into the three groups above with each serving as a frame. Respondents were then selected from the frames by simple random sampling using the ratio of 50:25:1.

Those who accepted to participate and signed an informed consent form were recruited.

### Informed consent process

Information on cervical cancer and cervical cancer screening were given to the women who were selected from the sample frame by research assistants as part of the informed consent process before signing the consent form. The study questionnaires were administered thereafter.

### Data collection

A semi structured questionnaire containing both closed and open ended questions specifically designed for the study was used for data collection. The questionnaire was pretested among 25 patients for comprehensibility, appropriateness of language, sensitivity of questions and average duration of administration. The feedback received after this process was used to modify and finalize the study questionnaire.

Information on sociodemographic characteristics, knowledge about cervical cancer, cervical cancer screening, previous screening history and personal perception of risk of developing cervical cancer were obtained from respondents who consented to be in the study. The questionnaire further enquired the willingness of the women to accept cervical cancer screening if offered. Those who showed willingness were asked to register their names with a trained counselor as indication of acceptance. The questionnaires were administered in English by trained research assistants. For low literates, the interview was conducted in their local dialect by trained research assistant who could communicate in that language.

### Variable definitions

· Age

The women’s age at their last birth day.

· Tribal group

The women were asked to state their tribe of origin. However during analysis the minority tribes in north and south were coded together because of their socio-cultural similarities and their small numbers.

· Religion

The two commonest religions in the country, Christianity and Islam were listed and non Christians and non Moslems were asked to mark others and specify their religion thereafter.

· Educational level completed

The women were asked to choose from options of from none, primary, secondary and tertiary.

· Marital status

Was determined by the question: How will you identify your marital status? Options listed were: Married, Not married and Widow.

· Living children

Was determined by the question: How many of your children are alive? They were expected to write the exact number of their children currently alive. However during analysis this response was dichotomized into two of Yes and No, where Yes denotes having at least one child alive and No denotes none.

· Duration of HIV disease

Respondents were asked to state the number of months elapsed since they were diagnosed HIV positive. This was dichotomized to ≤12 months, 13–36 months and >36 months.

· Awareness of cervical cancer and testing

Refers to an affirmative answer to two questions; 1. Have you heard of cervical cancer? 2. Are you aware of the test used to screen for cervical cancer?, and in addition explained in their own language what they know about them.

· Ever tested for cervical cancer

The women were asked whether they ever had tested for cancer of the cervix. The options were Yes or No.

· Self-risk assessment

Respondents were asked to assess their level of risk of having cervical cancer. They were expected to choose the alternatives; high, low or none.

· Willingness to screening for cervical cancer

Refers to women who answered Yes to the question “Do you want to be screened for cervical cancer “in the study questionnaire.

· Acceptance to screen for cervical cancer in future

Refers to women who answered yes to the question “Do you want to be screened for cervical cancer “and in addition registered their name with the counselor for screening. However respondent who were not willing to screen initially but eventually registered their name with counselor were re-categorized into the group of “ future acceptance of cervical cancer screening” group.

### Data analysis

The obtained information were coded, entered into the computer and analyzed using the SPSS version 19.0 (SPSS Inc. Chicago, IL) statistical packages. The main outcome variable was acceptance of cervical cancer screening. Univariate analysis using the Chi-square statistic was performed to identify factors associated with the acceptance of cervical cancer screening. Multivariate logistic regression was used to identify independent factors for acceptance of cervical cancer screening. Variables were entered into the model if their *P* value on univariate analysis was 0.25 or less. Odds ratio (OR) or adjusted OR (AOR) and their 95% confidence intervals (CI) were used to measure strength of associations. A P-value (two-tailed test) of <0.05 was considered significant. Acceptability rate was calculated as the number of women who accepted to screen out of the number of women who participated in the study.

### Ethical issues

Approval for the study was obtained from the Institutional Review Board, Nigerian Institute of Medical Research, Lagos Nigeria. Written informed consent was obtained from all women, before interview. The women were approached after the receipt of care so as to ensure that they were not coerced into the study. The clinic patients are organized into an independent support group of people living with HIV (Positive Life Organization of Nigeria) that ensures that patient’s rights are not violated.

## Results

A total 1675 women were invited to participate in the study; of which 1558 accepted and received the questionnaire. Forty-one of these were either not returned or returned uncompleted (response rate of 90.6%; 1517).

### Characteristics of the respondents

Table 1 shows the sociodemographic characteristics of the 1517 respondents that returned completed questionnaires. Most of the respondents were Christians (56.0%) and infected through heterosexual contact (79.6%). The mean age of the women was 31 ± 7 years (range 18–57). The majority of the respondents were between ages 30 to 49 (75.0%). Yoruba (37.1%) and Igbo (37.9%) were the predominant tribes of the respondents. One thousand two hundred and fifty seven (82.9%) of 1517 women completed at least secondary level education, 223(14.7%) completed primary and 37 (2.4%) women had no formal education. On the average the respondent had 2 living children and been diagnosed HIV positive for 39.5 months (range 0–119 months).

**Table 1:** The Sociodemographic characteristics of the respondents, Lagos, Nigeria April 2011 (n = 1517)

**Sociodemographic characteristics**	**Number of respondents (%)**
**Age (Years)**	
· Less than 20	16(1.1)
· 20 – 29	316(20.8)
· 30 – 39	797(52.5)
· 40 – 49	341(22.5)
· 50 and above	47(3.1)
**Religion**	
· Christianity	849(56.0)
· Islam	664(43.8)
· Others	4(0.2)
**Marital Status**	
· Married	923(60.8)
· Never Married	449(29.6)
· Widow	145(9.6)
**Ethnic Group**	
· Yoruba	568(37.1)
· Igbo	579(37.9)
· Hausa	80(5.2)
· Southern Minority	224(14.7)
· Northern Minority	57(3.7)
· Others	13(5.1)
**Educational level completed**	
· None	37(2.4)
· Primary	223(14.7)
· Secondary	776(51.2)
· Tertiary	481(31.7)
**Number of Living Children**	
· 0 – 1	764(50.4)
· 2 – 4	675(44.5)
· 5 and above	78(5.1)
**Duration of HIV disease (Months)**	
· Less than 13	417(27.5)
· 13 – 36	422(27.8)
· Greater than 36	678(44.7)
**HIV risk of transmission**	
· Heterosexual Contact	1208(79.6)
· Blood and Blood product	233(15.4)
· Others	78(5.0)

### Cervical cancer awareness and acceptance of testing

The cervical cancer awareness, self-risk assessment, screening knowledge and practice is shown in Table 2. Eight hundred and fifty three respondents (56.2%) were aware of cervical cancer and only 523 (34.5%) respondents were aware of cervical cancer screening/test. Only 143 (9.4%) respondents had ever tested for cervical cancer. The majority of the respondents assessed their risk of developing cervical cancer as low (68.2%), however 79.8% (1210) respondents accepted to take cervical cancer screening/test.

**Table 2:** Cervical cancer awareness, self-risk assessment, screening knowledge and practices among HIV infected Nigerian women Lagos, Nigeria April 2011 (n = 1517)

**Variables**	**No of respondents (%)**
**Aware of cervical cancer?**	
· **Yes**	853(56.2)
· **No**	664(43.8)
**Aware of cervical cancer testing?**	
· **Yes**	523(34.5)
· **No**	994(65.5)
**Ever Tested for Cervical Cancer?**	
· **Yes**	143(9.4)
· **No**	1374(90.6)
**Cervical cancer self-risk assessment**	
· **Low**	1034(68.2)
· **High**	483(31.8)
**Acceptance of cervical cancer screening?**	
· **Accepted**	1210(79.8)
· **Rejected**	307(20.2)

### Predictive factors for acceptance of testing

To determine predictors of acceptance of cervical cancer screening, we compared the sociodemographic, cervical cancer awareness status and self-risk assessment data with cervical cancer screening acceptance status among the respondents (Table 3). Differences in accepting to screen for cervical cancer were found for various variables. At univariate analysis, a greater percentage of women who accepted to take the test had more than secondary education (OR: 1.4; 95% CI:1.03 -1.84), no living children (OR:1.5;95% CI:1.06-1.99*),*diagnosed HIV positive within one year (OR:1.49;95% CI:1.11-2.02) and were aware of cervical cancer (OR:1.53;95% CI:1.19-1.96). After controlling for potential confounding variables of age, educational status, duration of HIV diagnosis and awareness of cervical cancer in a multivariate logistic regression model, it showed that women who had more than secondary education were 1.4 times likely to accept to take the test as compared with those who had less than secondary education (OR = 1.4; 95% CI:1.03-1.84). Respondents who had no living children were one and half times more likely to take the test than their counterparts with living children (OR: 1.5; 95% CI: 1.1-2.0). Women whose HIV diagnosis were made within one year were one and half times more likely to take the test than the respondents whose diagnosis were made over 3 years (OR:1.5; 95% CI:1.1-2.0). The women who were aware of cervical cancer and cervical cancer testing were 2 times more likely to take the test than those who were not aware of the disease and test (OR:1.5; 95% CI: 1.2-2.0). However, religion, ethnic grouping, marital status, self-risk assessment status and previous cervical cancer screening experience were found not to be associated with test uptake.

**Table 3:** Association between acceptance of cervical cancer screening, sociodemographic status of the respondents, cervical cancer awareness, self-risk assessment and history of previous cervical cancer screening (n = 1517)

**Variables**	**Accepted cervical cancer screening**		**95% CI Crude OR**	**95% CI Adjusted OR**
	**Yes (%)**	**No (%)**		
**Age (years)**				
· **Less than 25**	54(4.5)	12(3.9)	0.89(0.46-1.71)	0.87(0.46-1.68)
· **25-34**	572(47.3)	149(48.5)	1 (ref)	1 (ref)
· **≥ 35**	584(48.2)	146(47.6)	1.04(0.81-1.35)	1.04(0.80-1.34)
**Tribal Group**				
· **Yoruba**	414(34.2)	109(35.5)	1.01(0.84-1.48)	1.10(0.49-2.48)
· **Igbo**	505(41.7)	127(41.4)	1 (ref)	1 (ref)
· **Northern Tribes**	113(9.3)	23(7.5)	0.97(0.66-1.40)	0.97(0.67-1.42)
· **Southern Minority Tribes**	176(14.5)	46(15.0)	0.71(0.36-1.40)	0.78(0.29-2.08)
**Religion**				
· **Christianity**	676(55.9)	173(56.4)	1 (ref)	1 (ref)
· **Islam**	532(44.0)	132(43.0)	0.99(0.77-1.27)	0.93(0.68-1.28)
· **Others**	3(0.1)	1(0.3)		
**Educational level completed**				
· Less than Secondary	206(17.0)	54(17.6)	1.31(0.87-1.87)	1.20(0.83-1.80)
· Secondary	605(50.0)	171(55.7)	1 (ref)	1 (ref)
· Greater than Secondary	399(33.0)	82(26.7)	1.43(1.03-1.84)	1.31(1.02-1.83)^a^
**Marital status**				
· Married	725(59.9)	198(64.5)	1 (ref)	1 (ref)
· Not married	485(40.1)	109(35.5)	0.82(0.63-1.07)	0.90(0.68-1.18)
**Living children**				
· **Yes**	875(72.3)	243(79.2)	1 (ref)	1 (ref)
· **No**	335(27.7)	64(20.8)	1.45(1.06-1.99)	1.62(1.31-1.89) ^b^
**Duration of HIV disease**				
· **<**13 months	318(26.3)	99(32.2)	1.49(1.11-2.02)	1.50(1.1-1.98)^c^
· 13-36 months	331(27.4)	91(29.6)	1 (ref)	1 (ref)
· ≥13 months	561(46.4)	1179(38.1)	1.32(0.97-1.79)	1.31(0.97-1.78)
**Aware of cervical cancer**				
· **Yes**	706(58.3)	147(47.9)	1.53(1.19-1.96)	1.49(1.15-1.95)^d^
· **No**	504(41.7)	160(52.1)	1 (ref)	1 (ref)
**Ever tested for cervical cancer**				
· **Yes**	118(9.8)	25(8.1)	1.22(0.78-1.91)	1.23(0.71-1.22)
· **No**	1092(90.2)	282(91.9)	1 (ref)	1 (ref)
**Self-risk assessment of cervical cancer**				
· **High**	381(31.5)	102(33.2)	1 (ref)	1 (ref)
· **Low**	829(68.5)	205(66.8)	1.08(0.82-1.43)	1.10(0.93-1.39)
· **None**	0(0.00)	0(0.00)		

Note: Potential confounders adjusted at multivariate logistic regression analysis.

a. Adjusted for age, time elapsed since HIV diagnosis and awareness of cervical cancer.

b. Adjusted for age, marital status and time elapsed since HIV diagnosis.

c. Adjusted for age, educational status, and awareness of cervical cancer.

d. Adjusted for age, educational status and time elapsed since HIV diagnosis.

### Reasons for non-acceptance of cervical cancer screening test

Of the 1517 that returned completed questionnaire, 307(20.2%) did not register their name with the counselor, indicating that they are not ready to undergo cervical cancer screening. While (92.5% women of the 307 women that were not ready to be screened for cervical cancer gave various reasons for their action (See Table 4), 7.5% had no reason. The most common reason for refusal was the anticipated high cost of the test (35.2%). Other reasons were religious denial (14.0%), the need to obtain partner’s approval (12.4%), anticipated long waiting time (12.7%), pregnant/recently delivered (10.7%) and fear of test outcome (4.2%).

**Table 4:** Reason for non-acceptance of cervical cancer screening among the respondents

**Reason**	**n(%)**
Cost of test related issues	108(35.2)
Religious denial	43(14.0)
Requires partners permission	38(12.4)
Time to take the test/ long waiting time	39(12.7)
“Am pregnant/Recently delivered”	33(10.7)
“Am afraid to take the test”	13(4.2)
Taken the test before	9(2.9)
Had surgery of the vulva	1(0.3)
No reason	23(7.5)
Total	307(100.0)

## Discussion

This study showed a relatively high cervical cancer screening acceptance rate of 79.8% despite a moderate level of awareness of cervical cancer and it’s testing among the HIV positive women. Having a tertiary education, no living child, recent HIV diagnosis and awareness of cervical cancer were found to be associated with acceptance of cervical cancer screening. The level of awareness of cervical cancer as a disease (56.2%) and its testing (34.5%) among the respondents in this study though similar to findings from some previous studies [12,19,20] is lower than the finding in two recent studies conducted in our environment in 2009 and 2011 [21,22]. One would have expected that the present study being among a group at risk for cervical cancer, that the awareness level would have been much higher. Secondly, as our study was conducted after the two most recent studies [21,22], we were expecting at least a modest increase on awareness level due to the introduction of cervical cancer programme by the Government of Nigeria. A plausible reason for the lower awareness in this study could be the lower educational attainment of the respondent in this study compared to the studies by Bukar et al [21] and Dim et al. [22]. Supporting the above assertion of the positive role of educational attainment to cervical cancer awareness and test uptake is the finding in this study in which women with tertiary education were 1.4 times likely to take the test than women with lower qualification. The finding also underscores the importance of education in increasing cervical cancer awareness and screening uptake. A more intensified health education on cervical cancer and testing should be introduced in our programme and other HIV programmes in other to increase the awareness of cervical cancer and screening among these at risk group. In addition, the abysmally low “ever tested for cervical cancer” response (9.4%) in this study confirms the poor state of cervical cancer prevention and control in Nigeria. This is one of the reasons for the unacceptable morbidity and mortality associated with cervical cancer in Nigeria.

Puzzling, however in this study is the obvious disconnect between low awareness of cervical cancer and screening, high- low self-risk assessment of self to have cervical cancer (68.2%) and the high test acceptance rate (79.8%) among the respondents when compared to the acceptance rate of 44.0% reported by McKenzie et al. among HIV positive Kenyan women [23]. Although the Kenyan study restricted their enrollment to only women aged 30–39 years compared to this study with no age restriction, the main reason may be the almost absolute trust and relationship that exists between HIV positive clients and their primary Physician. The daily counseling and health talk by Counselors and Nurses obviously has a positive role in the high rate. To further strengthen the cervical cancer prevention programme, identification of the reason for the high rate of 79.8% among our cohort calls for an in-depth study using qualitative research methodology.

The present study apart from determining the level of awareness and acceptability of cervical cancer screening in future, determined the risk factors associated with future cervical cancer test uptake with the aim of using the information as counseling and advocacy tool. Awareness of cervical cancer screening and testing was found to be independently associated with the acceptance of cervical cancer screening. Respondent who were aware of cervical cancer as a disease were 1.5 times likely to accept the screening than those who are not aware. With awareness, one is likely to know the benefits of the test and thus opts for it. Awareness and education has been shown to improve the health seeking behaviour of individuals as it relates to uptake of cervical cancer services including testing [21-24]. The finding of respondents with tertiary education being 1.4 times likely to take the test compared to their peers with lower educational attainment support the above assertion. Educational attainment has been shown to be associated with acceptance of reproductive services and this is not only linked to women’s empowerment of making decision but ability to pay for these services without recourse to their partners [22]. Education is also linked with patient's self-perception of cervical cancer and understanding health education and counseling that goes on with it [21].

Another surprise finding in this study is that women who are relatively new in our programme are almost 2 times more likely to accept screening than women who have been in the programme for a longer time. Though the other previous studies did not evaluate this variable, making it difficult for comparison, evidence has shown that health seeking behaviour of individuals are better during symptomatic illness than if there is no symptom [25]. Also as opportunistic illness disappears and quality of life improves with antiretroviral therapy, other life events become more important than performance of test that the usefulness is not immediately obvious. Health education and counseling among HIV positives should therefore increase rather than decrease as the client’s health get better. The cervical cancer test acceptance in this study was found to be 1.5 times higher in respondent who do not have any living child compared to their peers with living children. The higher acceptance rate among the respondents with no living children may be due to their expected better knowledge as a result of their higher educational attainment. It is therefore expected that well-educated and knowledgeable persons will opt for a healthier life style of cervical cancer screening.

In as much as one may be tempted to accept the acceptance rate of 79.8% as good, looking at the other side of the flip- a refusal rate of 20.2% is unacceptably high especially for a lethal illness among a group of person at increased risk for the disease. If we must reduce the morbidity and mortality associated with cervical cancer, we should reduce the refusal rate to single digit if not to 0%. With the reduction of refusal rate in mind, we determined the reasons for test refusal among the respondents. The anticipated cost of testing (35.2%), religious denial (14.0%), need to obtain partners’ permission (12.4%) and long waiting time (12.7%) were found to be main reasons for non-acceptance of the test (see Table 4). It is rather surprising that respondents attributed cost as the major reason for their refusal to take the test, as cervical cancer screening is free at the centre. The obvious explanation might be that either they are unaware that it is free just like other HIV services or the cost they referred to may be cost related to transportation and loss of revenue as a result of having to spend almost a whole day at the clinic. Supporting the last reason is the fact that long waiting time was also a common reason for refusal. While transport cost can be reduced by taking services closer to client homes, it could also lead to decentralization and reduction in waiting time. Tasks shifting is the ultimate strategy to solving the challenge of long waiting time at the clinic. Evidence has consistently shown that delegation of tasks, whether from doctors to non-physician health workers especially the midwives can lead to improvement in access, coverage and quality of health services at comparable or lower cost than traditional delivery models [14,15,26]. In addition, it will reduce the workload on the physicians who are already overwhelmed from providing HIV services. We have successfully task shifted some HIV services to other health care workers and thus task shifting cervical cancer screening will build on the existing task shifting model. In addition, scheduling the cervical cancer screening during routine HIV clinic consults and drug refill will ensure that all the women who want to be tested do not spend extra days and time accessing this service. This will ultimately increase the acceptance rate. The other reasons for refusal ‘need partner’s permission’ and religious denials should be addressed urgently. The government through advocacy and possibly legislation needs to urgently address the encroachment of the religious miscreants into health without providing health infrastructure like the traditional mission hospitals. This encroachment has dire consequences as the poor and vulnerable groups now believe these men to a higher extent than the health care workers [16]. It is therefore not a surprise finding in this study that the second most common reason for refusal of the test was related to client’s religious belief and teaching. Some clients think that they cannot actually have cervical cancer because of their religious belief. This belief is as a result of activities of some religious leaders that diseases are only from the devil and children of God do not have diseases [16,17].

Low status of women and lack of autonomy in taking decisions should be seriously addressed. The current situation where a significant number of women cannot take decision whether to participate in a lifesaving test as a result of sociocultural and religious impediment is regrettable. Government of Nigerian and Ministry of Women Affairs in particular should focus on it and bring it to the front burner.

A possible limitation of the study was possible biased answers from the patient to please researchers who are their primary care provider. This could have overestimated the acceptance rate of cervical cancer test. We tried to eliminate getting bias answers by only administering the questionnaire to the patients after they had completed their routine HIV care for the day. In addition the questionnaire and informed consent process were administered by peer counselors who are HIV positives and members of the clinic support group.

Finally, it is important to state that educating women on the importance of cervical cancer screening and providing cervical cancer screening services is just not enough. Even more important is the provision of accessible and affordable treatment services. Government of Nigeria at all levels are urged not stop at creating awareness and screening services, but endavour to provide affordable and accessible treatment services.

## Conclusion

The study shows that HIV positive women in our centre are willing to be screened for cervical cancer and thus service integration will only strengthen rather than disrupt the existing HIV services. The refusals as a result of anticipated cost, long waiting time and spousal approval need to be urgently addressed through advocacy and public enlightenment.

## Competing interests

The authors declare that they have no competing interests.

## Authors’ contributions

OE conceived, designed the study, supervised data collection, analyzed the data, drafted the paper and approved the final version. CG co-supervises the data collection and entry, cleaned the entered data, reviewed result of data analysis and contributed to the drafting of the paper. KO contributed to the conception, modified the original concept and design, supervised the study, reviewed and contributed to the manuscript drafts. PO contributed to the conception, supervised the study, reviewed and contributed to the manuscript drafts. All authors read and approved the final manuscript.

## Pre-publication history

The pre-publication history for this paper can be accessed here:

http://www.biomedcentral.com/1471-2458/13/46/prepub
